# Supplementary material for: Content-rich biological network constructed by mining PubMed abstracts
Source: BMC Bioinformatics. 2004 Oct 8;5:147. doi: 10.1186/1471-2105-5-147 (PMC528731; doi:10.1186/1471-2105-5-147)
Supplement: Additional File 2 — The original results of the above study (non-essential files are deleted to keep the file size under the limit set by BMC bioinformatics). [file 1471-2105-5-147-S2.bz2 › chilibotAdditionalFile2/dip20/338ID9228058E4140/html/TNFSF6_TNFRSF6.html]

 


 **TNFSF6** and **TNFRSF6** 
  
Found 4489 abstracts in PubMed, retrieved 20.  
 

 What does Google say? 
 PDF only 
| .edu only 

---

**Interactive relationship** (e.g. stimulation, inhibition, etc)

**Stimulatory relationship**- Furthermore, POH treatment induced a transient G 2 M arrest, enhanced the expression of the membrane bound form of the Fas  [ **TNFRSF6** ]  ligand  [ **TNFSF6** ]  and sensitized the cells to Fas  [ **TNFRSF6** ]  mediated apoptosis.  Ref: 12886519 Prostate, 2003
**Neutral relationship**- Apoptosis did not require prior exposure to interleukin 2, nor was it the consequence of CD178  [ **TNFSF6** ]  CD95  [ **TNFRSF6** ]  or TNF TNF R interactions, and was therefore clearly different from activation induced cell death.  Ref: 12893746 BloodBlood, 2003
- KG1a cells CD34 38 express FAS  [ **TNFRSF6** ]  and TRAIL tumour necrosis factor related apoptosis inducing ligand receptors but are resistant to FAS  [ **TNFRSF6** ]  ligand  [ **TNFSF6** ]  and TRAIL APO2 L apoptosis antigen 2 ligand induced apoptosis.  Ref: 12877678 Br J HaematolBr J Haematol, 2003
- Alterations in expression or shedding of membrane and soluble CD95L  [ **TNFSF6** ]  are associated with numerous diseases, and underscore the pathophysiological relevance of the CD95  [ **TNFRSF6** ]  CD95L  [ **TNFSF6** ]  system.  Ref: 12894217 Cell Death DifferCell Death Differ, 2003

**Non-interactive relationship** (e.g. studied together, co-existance, homology, etc.)

- 2 Fas  [ **TNFRSF6** ]  mRNA and FasL  [ **TNFSF6** ]  mRNA were not expressed in the controls.  Ref: 12887767 Zhonghua Yi Xue Za Zhi, 2003
- ECP treated lymphocytes of chronic graft versus host disease patients undergo apoptosis which involves both the Fas  [ **TNFRSF6** ]  FasL  [ **TNFSF6** ]  system and the Bcl 2 protein family.  Ref: 12883827 Arch Dermatol ResArch Dermatol Res, 2003
- In conclusion, tolerance induced by TGF beta treated APC appears to be associated with deletion of antigen specific T cells involving the Fas  [ **TNFRSF6** ]  FasL  [ **TNFSF6** ]  pathway.  Ref: 12882832 Int Immunol, 2003
- This study presents a non pharmacological and spatially limited approach for protection of VPC grafted in the CNS against destruction by host immune responses.Murine fibroblast derived VPC expressing herpes simplex virus type I thymidine kinase HSV tk were genetically modified to co express a human Fas  [ **TNFRSF6** ]  ligand  [ **TNFSF6** ]  CD95L  [ **TNFSF6** ]  deletion mutant DeltaFasL resistant to enzymatic cleavage and shedding.  Ref: 12888123 Life Sci, 2003
- Thus, WT CFTR expression promotes a rapid expression of CD95  [ **TNFRSF6** ]  CD95  [ **TNFRSF6** ]  ligand  [ **TNFSF6** ]  and apoptotic response to P. aeruginosa infection.  Ref: 12878584 Am J Respir Cell Mol Biol, 2003
- Overexpression of Bcl 2 may inhibit the Fas  [ **TNFRSF6** ]  FasL  [ **TNFSF6** ]  induced apoptosis.  Ref: 12887767 Zhonghua Yi Xue Za Zhi, 2003
- Immediately after isolation, there was no difference in Bcl 2 or bax expression among the four different samples, or in Fas  [ **TNFRSF6** ]  and FasL  [ **TNFSF6** ]  mRNA.  Ref: 12883827 Arch Dermatol ResArch Dermatol Res, 2003
- The level of Bcl 2 remained largely unchanged, but the Bax, Fas  [ **TNFRSF6** ] , and FasL  [ **TNFSF6** ]  expression showed up regulation.  Ref: 12880947 Biochim Biophys Acta, 2003
- and expression of perforins, Fas  [ **TNFRSF6** ]  ligand  [ **TNFSF6** ] , and other functional markers.  Ref: 12891535 Gastroenterology, 2003
- A liver tolerates a portal antigen by generating CD11c cells, which select Fas  [ **TNFRSF6** ]  Ligand  [ **TNFSF6** ]  Th2 cells via apoptosis.  Ref: 12883484 Hepatology, 2003
- It also induces fas  [ **TNFRSF6** ]  ligand  [ **TNFSF6** ]  expression, activates procaspase 8 and 9 cleavage, reduces mitochondrial membrane potential, and increases cell death as determined by propidium iodide staining.  Ref: 12876280 J Biol ChemJ Biol Chem, 2003
- Perforin, granzyme B, and Fas  [ **TNFRSF6** ]  ligand  [ **TNFSF6** ]  were only observed before IVIG administration.  Ref: 12877456 Ann Allergy Asthma Immunol, 2003
- Factors like cytokines, prostaglandine, microbes, MHC molecules, hormones, Fas  [ **TNFRSF6** ]  ligand  [ **TNFSF6** ] , heat shock proteins, phenotypes of dendritic cells and APCs, probably can be polarizing courses of eTLM pathway.  Ref: 12888311 Med Hypotheses, 2003
- While Fas  [ **TNFRSF6** ]  and FasL  [ **TNFSF6** ]  are involved in apoptosis of bystander cells, mitochondrial pathway is required for apoptosis of infected cells.  Ref: 12888173 Rev Med Interne, 2003
- Expression of mutant non cleavable Fas  [ **TNFRSF6** ]  ligand  [ **TNFSF6** ]  on retrovirus packaging cells causes apoptosis of immunocompetent cells and improves prodrug activation gene therapy in a malignant glioma model.  Ref: 12888123 Life Sci, 2003
- In conclusion, in patients affected by cGVHD, ECP induced apoptosis of lymphocytes with the involvement of both the Fas  [ **TNFRSF6** ]  FasL  [ **TNFSF6** ]  system and the Bcl 2 protein family.  Ref: 12883827 Arch Dermatol ResArch Dermatol Res, 2003
- To elucidate molecular events in the apoptosis, protein expressions of Bcl 2, Bax, Fas  [ **TNFRSF6** ] , and FasL  [ **TNFSF6** ]  were measured by Western blotting using specific antibodies in HL 60 cells.  Ref: 12880947 Biochim Biophys Acta, 2003
- Pseudomonal infection induced expression of CD95  [ **TNFRSF6** ]  and CD95  [ **TNFRSF6** ]  ligand  [ **TNFSF6** ] , a response that was also delayed in cells homozygous for mutant Cftr alleles.  Ref: 12878584 Am J Respir Cell Mol Biol, 2003
- Immunofluorescence staining together with ordinary and confocal microscopy confirmed the observations that procaspase 3 immunoreactivity was clearly identified in the cytoplasm and nucleus while Fas  [ **TNFRSF6** ]  ligand  [ **TNFSF6** ]  staining was localized to the cell surface and PARP immunoactivity to the nucleus, which were used as controls.  Ref: 12883646 Int J Mol Med, 2003
- The CD95  [ **TNFRSF6** ]  ligand  [ **TNFSF6** ]  is involved as a death factor in the regulation of activation induced cell death, establishment of immune privilege and tumor cell survival.  Ref: 12894217 Cell Death DifferCell Death Differ, 2003
- Similar processing occurs in TNF, LPS, Fas  [ **TNFRSF6** ]  ligand  [ **TNFSF6** ] , and thapsigargin Tg induced apoptosis.  Ref: 12885762 J Cell BiolJ Cell Biol, 2003
- Cell death was prevented and the ability to expand in vitro was restored by treatment with anti Fas  [ **TNFRSF6** ]  ligand  [ **TNFSF6** ]  FasL  [ **TNFSF6** ]  antibody.  Ref: 12882832 Int Immunol, 2003
- The expression of Fas  [ **TNFRSF6** ]  FasL  [ **TNFSF6** ]  has no effect on the gastric mucosa apoptosis related to WRS.  Ref: 12887767 Zhonghua Yi Xue Za Zhi, 2003
- CD95  [ **TNFRSF6** ]  ligand  [ **TNFSF6** ]  death factor and costimulatory molecule ? .  Ref: 12894217 Cell Death DifferCell Death Differ, 2003
- Administration of an antigen Ag per oral route leads to apoptosis of Ag specific CD4 T cells and to development of Th2 cells expressing Fas  [ **TNFRSF6** ]  ligand  [ **TNFSF6** ]  FasL  [ **TNFSF6** ]  in the liver.  Ref: 12883484 Hepatology, 2003
- OBJECTIVE To investigate the correlation between the expressions of apoptosis promoting gene Bax, apotosis inhibiting gene Bcl 2, and Fas  [ **TNFRSF6** ]  FasL  [ **TNFSF6** ]  in gastric mucosa in the course of stress ulcer.  Ref: 12887767 Zhonghua Yi Xue Za Zhi, 2003
- In situ hybridization was used to detect the mRNA expression of Bcl 2, Fas  [ **TNFRSF6** ] , and FasL  [ **TNFSF6** ] .  Ref: 12887767 Zhonghua Yi Xue Za Zhi, 2003
